# An Exploration of the Effectiveness of Different Intensity Protocols of Modified Constraint-Induced Therapy in Stroke: A Systematic Review

**DOI:** 10.1155/2023/6636987

**Published:** 2023-10-10

**Authors:** Pavlina Psychouli, Ioannis Mamais, Charalambos Anastasiou

**Affiliations:** Occupational Therapy Program, Department of Health Sciences, European University Cyprus, Cyprus

## Abstract

**Purpose:**

To examine the effectiveness of different modified Constraint-Inuced Therapy (mCIMT) protocol intensities on upper extremity motor function in adults with hemiplegia.

**Methods:**

A search was conducted in PubMed, Scopus, EBSCO, and Cochrane Library for articles published between April 2010 and December 2021. Only randomized controlled trials (RCTs) were included. Studies were excluded if they used a sample of less than five, mCIMT in combination with other therapy, and/or if they were not written in English. Methodologic quality was assessed using the Cochrane collaboration risk of bias tool–2.

**Results:**

Thirty-six RCTs with a total of 721 participants were included. Most researchers followed a moderate to low protocol intensity in terms of total treatment time and moderate to high intensity with regard to restriction time. Almost all of the upper limb motor function measures showed statistically significant improvements (*p* < .05) after mCIMT, irrespective of the protocol's intensity, but there was lack of high-quality studies. Statistically significant improvements did not always translate to clinical importance.

**Conclusions:**

Low-intensity CIMT protocols may result in comparable improvements to more intensive ones but caution has to be taken when drawing conclusions due to high risk of bias studies.

## 1. Introduction

Stroke incidence has raised since 2000 and is now the second leading cause of death worldwide. Globally, low- and middle-income countries present the highest rates of stroke prevalence [1]. In 2019, five leading risk factors for stroke were identified by the Global Burden of Diseases, Injuries, and Risk Factors Study, including high systolic blood pressure, high body mass index, high fasting plasma glucose, ambient particulate matter pollution, and smoking [2].

Manual dexterity is frequently impaired in stroke persons, resulting in long-term functional deficits [3]. According to the International Classification of Functioning, Disability, and Health (ICF) [4], it is expected that the severity of upper limb impairment may correlate with high dependence levels in activities of daily living, lack of participation, and low quality of life. As Raghavan [5] described, the functional consequences of stroke on the upper limb may be connected with learned nonuse, learned bad use, and unlearning or forgetting after stroke. The learned nonuse phenomenon was originally described by Taub et al. [6], who argued that limited use of the hemiplegic upper limb is rarely the direct result of brain insult but rather the effect of a gradually developed behavioral neglect. Constraint-induced movement therapy (CIMT) was introduced as a treatment method aiming at reversing learned nonuse. The original protocol consisted of three basic elements: (1) 6 hours of task-oriented intensive therapy, for a total of 2-3 weeks, (2) a “transfer package” facilitating transfer gains to the real world, and (3) restriction of the unaffected upper limb for 90% of the patient's waking hours [7].

Despite the positive effects demonstrated for CIMT [8–12], this treatment method was initially considered difficult to implement clinically by Page et al. [13]. Since then, researchers have applied different modified versions of the treatment (modified constraint-induced movement therapy or mCIMT), highly variable but typically characterized by reduced daily restraint and treatment time. As a matter of fact, reviewing of the literature reveals that there is more published research on mCIMT than the original CIMT, perhaps reflecting researchers' response to clinical demand.

Many studies have shown the effectiveness of mCIMT and even its superiority against other therapeutic methods, usually traditional care [14–16]. However, mCIMT is not represented by a single protocol. Previous systematic reviews have explored either the effectiveness of CIMT and mCIMT together [8, 11] or included all mCIMT protocols without differentiating between various intensities [17]. In addition, most of the previous systematic reviews have examined the effectiveness of CIMT or mCIMT in comparison to other treatment rather than within the experimental group [8, 17]. Fleet et al. [18] focused their review on protocols that were offered in 3 sessions per week, for 10 weeks, but the daily session and restriction time were not controlled for. Nijland et al. [19] compared between two different intensities of mCIMT in acute and subacute stroke, but their conclusions have to be cautiously interpreted due to the small number of included studies. To our knowledge, there has been no published review up until now comparing the before-after effect of mCIMT.

This review is aimed at exploring for the first time the relative effectiveness of different intensities of mCIMT protocols by dividing studies into three intensity groups, corresponding to the overall treatment time and the splint-wearing time set by each research group. Therefore, the results of clinical tests of upper limb motor function in persons with stroke were reviewed before and after application of mCIMT.

## 2. Methods

This systematic review was conducted according to the preferred reporting items for systematic reviews and meta-analysis (PRISMA) guidelines [20].

In order to explore the relative effectiveness of various intensity mCIMT protocols, studies were categorized by our research team, according to the overall treatment time as "low" (less than 20 hours), "moderate" (21-39 hours), or "high" (more than 40 hours) intensity, and according to the daily splint-wearing time as "low" (≤ 3 hours/day), "moderate" (4-9 hours/day), and "high" (≥ 10 hours/day).

### 2.1. Study Selection

#### 2.1.1. Inclusion Criteria

The research question was formulated using the PICOS framework, which identifies (1) the population; (2) the intervention; (3) comparison or control groups; (4) outcomes; and (5) study design, as follows:

*P:* adults diagnosed with stroke.

*I:* mCIMT.

*O:* outcome measures related to upper limb motor function.

*S:* randomized controlled trials (RCTs).

Therefore, articles were included if they were randomized controlled trials, satisfying the following criteria: having investigated at least one modified protocol of CIMT, providing data before and after the intervention; having applied treatment to adults (people 18 years and older), following stroke; having included outcome measures related to upper limb motor function; and having been written in English. The last published systematic review and meta-analysis that presented the comparison between mCIMT and traditional rehabilitation included studies from inception to April 10, 2010 [17]. Therefore, this systematic review included studies published from April 10, 2010, to December 31, 2021.

#### 2.1.2. Exclusion Criteria

Exclusion criteria ensured that the systematic review consisted only of research studies pertaining to the study population and mCIMT. Excluded studies comprised those which provided mCIMT in addition to any other intervention, with sample size less than 5 in the mCIMT group, unfinished studies, or studies that did not contain specific, numeric results (including grey literature). Additionally, kinematic analysis or brain physiology measures were excluded.

### 2.2. Data Sources

Two investigators (I.M. and C.A.) conducted a structured search of the following databases: PubMed, Scopus, EBSCO, and Cochrane Library having used the MeSH terms included in Table 1. There were no additional advanced search methods used for each of the databases listed.

### 2.3. Data Extraction

Two investigators (C.A. and I.M.) screened the abstracts identified in the database searches. C.A. applied the inclusion and exclusion criteria to the abstracts; if the information in the abstract did not meet the selection criteria, the study was excluded at this stage, and the reason for exclusion was recorded. Duplicate articles were removed. The remaining full-length articles were then retrieved and reviewed by C.A. to further determine that the study was in accordance with the selection criteria. The final decision was reached after the senior investigator (P.P.) had performed another review.

### 2.4. Classifying the Evidence and/or Determining the Level of Confidence and Recommendations

In order to assess the risk of bias, the Cochrane risk-of-bias tool for randomized trials (R*ο*B-2) was used independently by two researchers (I.M. and C.A). RoB-2 consists of five areas for RCT bias risk assessment. These five domains assess the risk of bias arising from the randomization process, deviations from intended interventions, missing outcome data, outcome measurements, and selection of reported results. During the assessment, each domain is rated as low, high, or having some concerns. Conflicting assessment ratings were resolved by consensus. At the end, each domain's results were calculated to reach an overall risk-of-bias rating of poor, fair, or good.

### 2.5. Data Synthesis and Analysis

Specific variables of interest were extracted from the studies and added to summary tables. Summary tables were then used to classify studies according to the following categories: treatment hours, splint wearing time, and outcome measures related to upper limb motor function. Out of these outcome measures, the following were identified as the most commonly used by researchers: The Fugl-Meyer assessment for upper extremity (FMA-UE), Motor Activity Log quality of movement (MAL-QOM) and amount of use (MAL-AOU), Wolf Motor Function Test performance time (WMFT-PT) and functional ability (WMFT-FA), and Action Research Arm Test (ARAT). Microsoft 365 Excel 2021 was used for data analysis and graph plotting.

### 2.6. Statistical Analysis

To satisfy the study's aims, pre and postintervention means were collected for the same group. The fact that there was lack of two independent group differences to compare at the same time prevented us from conducting a meta-analysis. Pre-post standardised mean differences (SMDs) are influenced by natural processes, personal characteristics, and settings, and these cannot be discerned from the effects of the intervention. In addition, pre-post SMDs should be avoided if the scores between pre and postintervention are not independent from each other [21].

### 2.7. Sensitivity Analysis

The influence of individual studies was examined by omitting low-quality studies to see the extent to which inferences depend on a particular study or group of studies (sensitivity analysis).

## 3. Results

### 3.1. Study Selection

**Figure 1:**
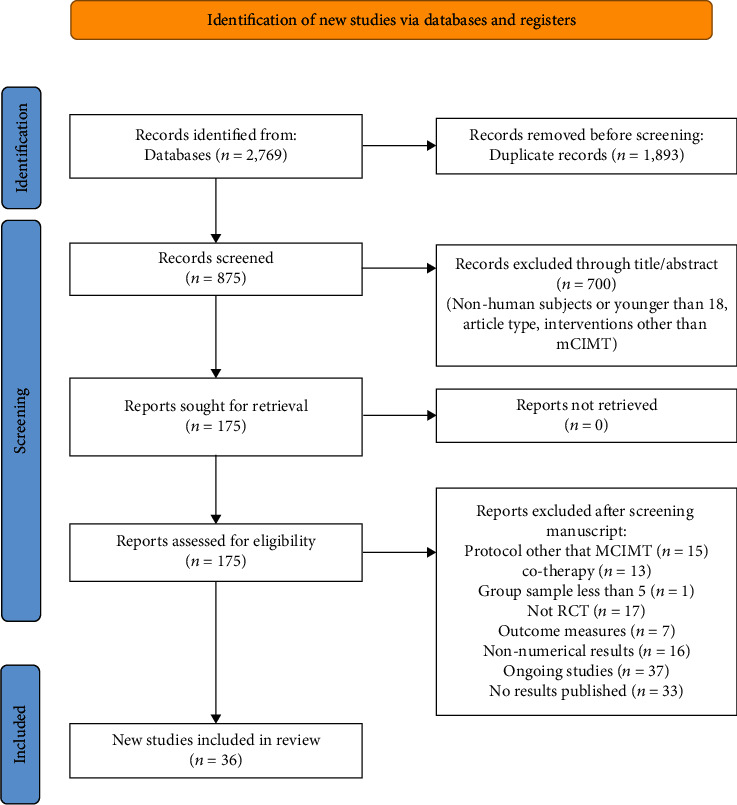
Flow chart diagram.

The search identified 2,769 papers, of which 875 full-text articles were assessed for eligibility. Out of these, 700 studies were excluded through title/abstract screening because they followed study designs other than RCTs or the population and interventions did not match the study's inclusion criteria. The second screening through reading of the whole manuscript resulted in exclusion of 53 studies due to intervention characteristics (i.e. they did not use mCIMT or used combination of treatments), outcome measures, and methodological issues (i.e. sample size). Seventy studies were excluded because they had not published any results until December 2021. One [22] additional study and 16 more that were listed as grey literature were excluded from the study because there were no numerical data, leading to the 36 studies of this systematic review (Figure 1).

### 3.2. Study and Participant Characteristics

The 36 studies included in this review were published between April 2010 and December 2021, in 21 countries: United States [23, 24], Australia [25–27], Brazil [28], Chile [29], China [30], Egypt [31], Germany [32], India [33–38], Iran [39], Israel [40], Italy [41], Japan [42], Jordan [43], Korea [44, 45], Netherlands [46, 47], Nigeria [48, 49], Norway [50, 51], Spain [52], Switzerland [53], Taiwan [54–57], and Turkey [58], with a total of 721 participants.

Participants ranged from 5 to 85 among the studies, while their mean age was between 46.2 and 65.3 years. The mean time since stroke ranged from 7.31 days to 58.8 months. The total intervention time varied from 2 weeks to 2 months. Nineteen studies [23, 24, 41–43, 46, 47, 49–51, 53, 25, 26, 29, 30, 32, 38–40] included a follow-up measurement, which ranged from 21 days to 1 year; 15 out of them measured follow-up in comparison to preintervention, while 9 studies measured follow-up in comparison to posttreatment values. A summary of the characteristics of the included studies is presented in Table 2.

Out of the 721 participants, 57 withdrew or did not comply with the study protocol. In more detail, 14 dropped out due to unrelated medical reasons [23, 26, 32, 41, 50, 51, 53], 5 for personal reasons [32, 58], 12 for uncooperativeness [24, 41], 14 lost contact or did not show up for examination [23–25, 38, 47, 51], 3 due to distant location [47, 50], 1 died [32], 2 declined treatment [51], 1 lost interest [23], 3 did not return the adherence log book [25], 1 missed too many sessions due to caregiver's schedule [24], and 1 refused the examination [30]. No adverse effects were reported by any study.

Five studies [32, 36, 42, 43, 49] used high-intensity protocols (total treatment time), 15 studies [23, 24, 54–58, 28–30, 33, 38, 39, 51, 53] used moderate intensity, and 16 studies [25, 26, 45–48, 50, 52, 27, 31, 34, 35, 37, 40, 41, 44] used low-intensity protocols. With regards to splint wearing time, 12 studies [23, 24, 51, 58, 25–27, 29, 36, 37, 41, 49] followed high-intensity restriction, 18 studies [30, 31, 44, 45, 47, 50, 54–56, 32–35, 38–40, 42] used moderate, and 6 studies [28, 43, 46, 48, 52, 53] used low-intensity restriction (Table 3).

### 3.3. Methodologic Quality Assessment

Out of the 36 studies, 29 were classified as “high” risk, according to RoB2 [24, 25, 36–41, 43, 44, 46, 47, 26, 48, 51–58, 27–30, 33–35], 3 as “some concerns” [31, 42, 50], and 4 as “low” [23, 32, 45, 49].  Table 4 shows the quality assessment of the studies, according to RoB2.

### 3.4. Outcome Measures

The outcome measures reviewed were the ones related to upper limb motor function, while graphs were plotted for the most commonly used ones. The latter included the FMA-UE, MAL (QOM and AOU), WMFT (PT and FA), and ARAT.

#### 3.4.1. Fugl-Meyer Assessment for Upper Extremity (FMA-UE)

**Figure 2:**
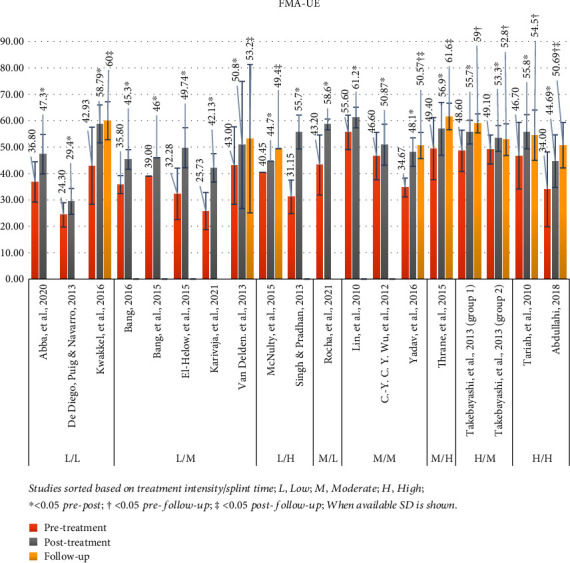
FMA-UE results. Studies sorted based on treatment intensity/splint time; L: low; M: moderate; H: high; ^∗^< 0.05 pre-post; †< 0.05 pre-follow-up; ‡< 0.05 post-follow-up; when available, SD is shown.

Eighteen studies used the FMA-UE [26, 28, 46–49, 51, 52, 54, 57, 31, 35, 37, 38, 42–45]. The mean value for pretreatment was 39.96 (range: 24.3-55.6). The mean value for posttreatment was 50.26 (range: 29.4-61.2). *P* value was <0.05 for all studies comparing before and after intervention except one [46]. Eight studies included follow-up assessment [26, 38, 42, 43, 46, 47, 49, 51]. The mean value for the follow-up was 54.64 (range: 49.4–61.6). *P* value was <0.05 for 4 studies [38, 42, 43, 49], measuring follow-up in comparison to pretreatment, and 5 studies measuring it in comparison to posttreatment [26, 38, 47, 49, 51].  Figure 2 presents a graphical display of FMA-UE data in relation to the various intensity mCIMT protocols used in each study.

#### 3.4.2. Motor Activity Log Quality of Movement (MAL-QOM)

**Figure 3:**
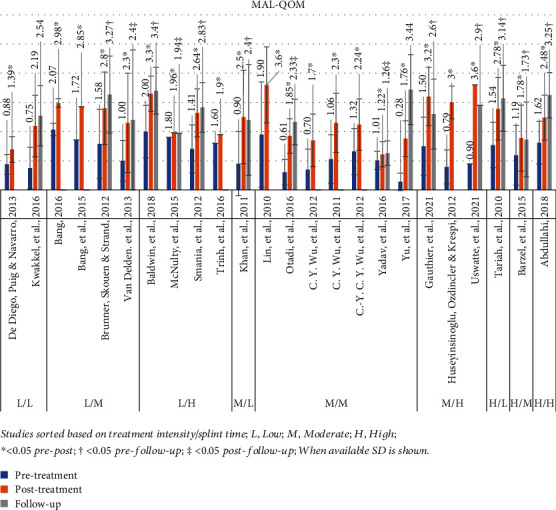
MAL-QOM results. Studies sorted based on treatment intensity/splint time; L: low; M: moderate; H: high; ^∗^< 0.05 pre-post; †< 0.05 pre-follow-up; ‡< 0.05 post-follow-up; when available, SD is shown.

Twenty-four studies used the MAL-QOM, presenting a mean pretreatment value of 1.3 (range: 0.3-2.1) [23, 24, 43–47, 49, 50, 52–54, 25, 55–58, 26, 27, 30, 32, 38, 39, 41]. The posttreatment mean value was 2.4 (range: 1.2-3.6), while the *P* value was <0.05 in 23 studies, which compared before and after intervention. Fifteen studies included a follow-up assessment, the mean value of which was 2.6 (range: 1.3-3.4) [23, 24, 46, 47, 49, 50, 53, 25, 26, 30, 32, 38, 39, 41, 43]. *P* value was <0.05 in 9 studies [23–25, 32, 38, 41, 43, 49, 50], which tested follow-up with reference to pretreatment, and in 4 studies [26, 38, 39, 47], which tested it with reference to posttreatment.  Figure 3 presents a graphical display of MAL-QOM data in relation to the various intensities of mCIMT protocols used in each study.

#### 3.4.3. Motor Activity Log Amount of Use (MAL-AOU)

**Figure 4:**
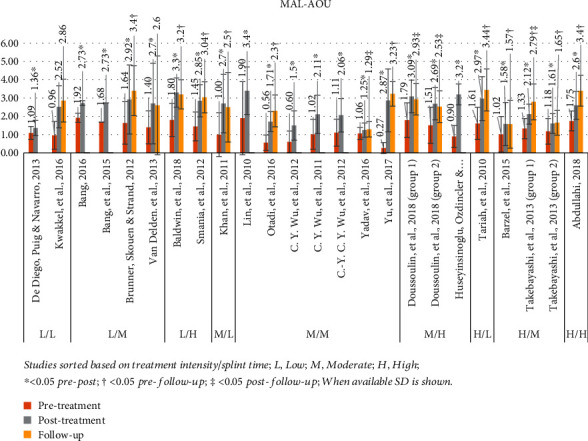
MAL-AOU results. Studies sorted based on treatment intensity/splint time; L: low; M: moderate; H: high; ^∗^< 0.05 pre-post; †< 0.05 pre-follow-up; ‡< 0.05 post-follow-up; when available, SD is shown.

The MAL-AOU was used in 22 studies [25, 29, 45–47, 49, 50, 52–56, 30, 57, 58, 32, 38, 39, 41–44]. Mean pretreatment value was 1.3 (range: 0.3-1.9) and posttreatment 2.4 (range: 1.3-3.4), with the *P* value <0.05 for 21 studies [25, 29, 45, 47, 49, 50, 52–57, 30, 58, 32, 38, 39, 41–44]. Fourteen studies included a follow-up assessment, with the mean value of MAL-AOU at 2.7 (range: 1.3–3.4) [25, 29, 47, 49, 50, 53, 30, 32, 38, 39, 41–43, 46]. Out of these studies, 9 showed a *P* value of <0.05 for pretreatment to follow-up [25, 30, 32, 41–43, 49, 50, 53] and 4 for posttreatment to follow-up [29, 38, 39, 42].  Figure 4 presents a graphical display of MAL-AOU data in relation to the various intensity mCIMT protocols used in each study.

#### 3.4.4. Wolf Motor Function Test Performance Time (WMFT-PT)

**Figure 5:**
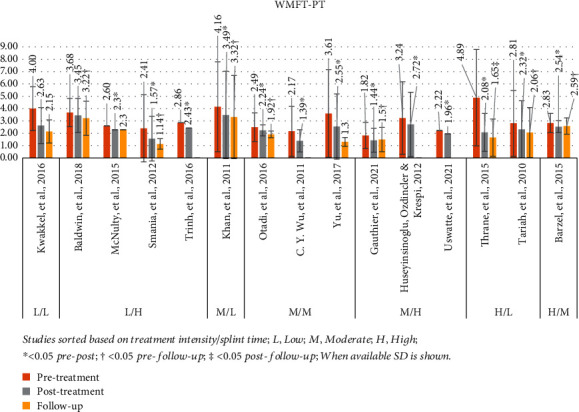
WMFT-PT results. Studies sorted based on treatment intensity/splint time; L: low; M: moderate; H: high; ^∗^< 0.05 pre-post; †< 0.05 pre-follow-up; ‡< 0.05 post-follow-up; when available, SD is shown.

The WMFT-PT was used in 15 studies, presenting a mean pretreatment value of 3.1 (range: 1.8-4.9) and posttreatment of 2.3 (range: 1.4-3.5) [23, 24, 46, 51, 53, 56, 58, 25–27, 30, 32, 39, 41, 43]. In 13 studies, the *P* value was <0.05 [23, 24, 53, 56, 58, 26, 27, 30, 32, 39, 41, 43, 51]. Follow-up assessment was included in 11 studies, with a mean value of 2.1 (range 1.1–3.3) [23, 25, 53, 26, 30, 32, 39, 41, 43, 46, 51]. Out of these studies, 8 showed statistically significant results at the follow-up, which for 6 studies was measured in comparison to pretreatment [23, 25, 32, 41, 43, 53] and for 2 in comparison to posttreatment [39, 51].  Figure 5 presents a graphical display of *WMFT-PT* data in relation to various intensity mCIMT protocols used in each study.

#### 3.4.5. Wolf Motor Function Test Functional Ability (WMFT-FA)

**Figure 6:**
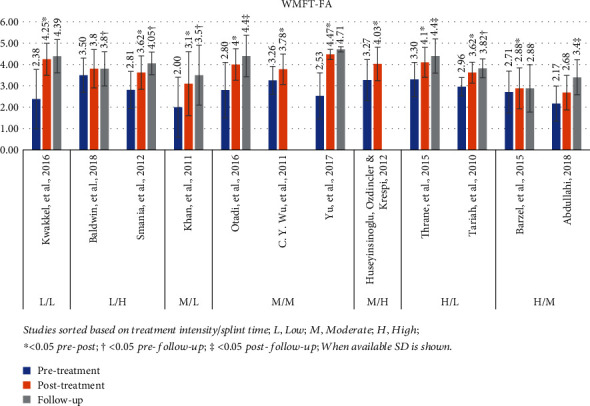
WMFT-FA results. Studies sorted based on treatment intensity/splint time; L: low; M: moderate; H: high; ^∗^< 0.05 pre-post; †< 0.05 pre-follow-up; ‡< 0.05 post-follow-up; when available, SD is shown.

The WMFT-FA was used in 12 studies, presenting a mean pretreatment value of 2.8 (range: 2.0-3.5) and posttreatment of 3.7 (range: 2.7-4.5) [25, 30, 56, 58, 32, 39, 41, 43, 46, 49, 51, 53]. In 10 studies, the *P* value was <0.05 [30, 32, 39, 41, 43, 46, 51, 53, 56, 58]. Follow-up assessment was included in 10 studies, with a mean value of 3.9 (range 2.9–4.7) [25, 30, 32, 39, 41, 43, 46, 49, 51, 53]. Out of these studies, 7 showed statistically significant results at the follow-up, which for 4 studies was measured in comparison to pretreatment [25, 41, 43, 53] and for 3 studies in comparison to posttreatment [39, 49, 51].  Figure 6 presents a graphical display of *WMFT-FA* data in relation to various intensity mCIMT protocols used in each study.

#### 3.4.6. Action Research Arm Test (ARAT)

**Figure 7:**
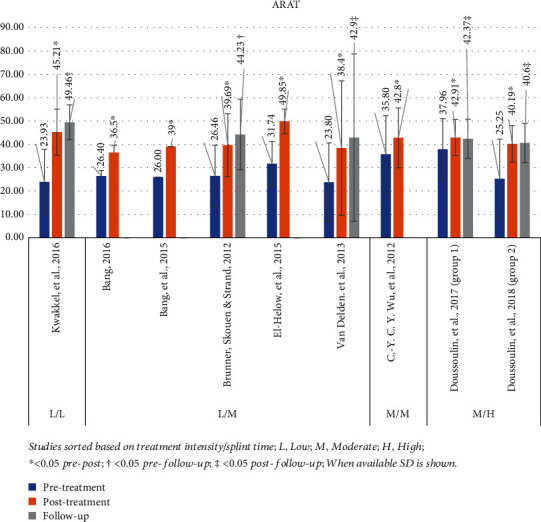
ARAT results. Studies sorted based on treatment intensity/splint time; L: low; M: moderate; H: high; ^∗^< 0.05 pre-post; †< 0.05 pre-follow-up; ‡< 0.05 post-follow-up; when available, SD is shown.

The ARAT was used in 8 studies, presenting mean pretreatment value of 28.6 (range: 23.8-38) and posttreatment of 41.6 (range: 36.5-49.9) [29, 31, 44–47, 50, 55]. The *P* value was <0.05 for all studies comparing before and after intervention. Follow-up assessment was included in 4 studies, with mean value of 43.9 (range 40.6–49.5) [29, 46, 47, 50]. *P* value was <0.05 for all studies, 2 of which measured follow-up in comparison to pretreatment [46, 50] and 2 in comparison to posttreatment [29, 47].  Figure 7 presents a graphical display of ARAT data in relation to various intensity mCIMT protocols used in each study.

Out of the 36 studies, 21 (*N* = 124) were focused on chronic patients [23, 24, 41–43, 45, 48, 52, 54–57, 25, 58, 26–29, 32, 36, 38]. Statistical insignificance was noted at the follow-up in four studies; one [42] used the FMA-UE, and the others [25, 26, 32] used the WMFT. One of these studies did not reach statistical significance at any point [32].

After sensitivity analysis to include only the low risk of bias studies, 4 out of the 36 studies were separately examined [23, 32, 45, 49]. Out of these studies, three focused on chronic [23, 32, 45] and one on acute stroke [49]. Statistical significance was shown in all outcome measures between pre and postintervention measurements and also between follow-up and preintervention. Statistical significance however does not coincide with clinical importance across all measures and all studies.

## 4. Discussion

This review identified 36 RCTs, corresponding to the inclusion criteria, and 721 participants in total. Almost all studies showed statistically significant improvements after mCIMT in the perceived arm motor function, as measured by MAL and arm motor impairment measures, in agreement to previous research [8, 17]. Some of the improvements were clinically important, and some were not, especially when follow-up measurements were compared to postintervention values. Nineteen studies [23, 24, 41–43, 46–48, 50, 51, 53, 25, 26, 29, 30, 32, 38–40] included at least one follow-up measurement and showed statistically significant retainment of improvements for most outcome measures. However, there were many studies in which there was misreporting of results or statistical significance.

Out of the 36 studies, 16 [25, 26, 45–48, 50, 52, 27, 31, 34, 35, 37, 40, 41, 44] used a low-intensity protocol, while only 5 studies [32, 36, 42, 43, 49] used what was classified as “high intensity” for this review. On the other hand, 18 studies [30, 31, 44, 45, 47, 50, 54–57, 32–35, 38–40, 42] included 4-9 hours of daily splint wearing time (moderate), while 12 studies [23, 24, 51, 58, 25–27, 29, 36, 37, 41, 49] included more than 10 hours daily restriction (high). This finding shows researchers' clear preference towards fewer hours of overall treatment, accompanied by more hours of hand restriction.

The results of this study show no major differences in the effectiveness of mCIMT protocols of different treatment intensities, and there is no support to the “more is better” notion. This comes in agreement with other studies [8, 11, 59], who also found that the dose of intervention did not influence the results. Fleet et al. [18] reviewed 15 studies of mCIMT that included 10-week intervention with frequency of 3 times per week; out of these studies, 10 used 30-minute sessions, and 5 used 60-minute sessions. Although the authors did not provide separate analysis between the two intensity protocols, graphical displays do not seem to support any substantial intensity effect. Similarly, Sterr et al. [59] found that using or not a constraint and having more or less shaping training did not significantly change the treatment outcome in low-functioning stroke persons. In fact, Nijland et al. [19] concluded that low-intensity protocols seem to be more beneficial when it comes to acute and subacute stroke.

However, suggesting that any protocol may be effective is an oversimplification, since it is widely acceptable and evidence based [60, 61] that intensity relates to neuroplasticity and directly translates to functional improvements. Reviewing the literature reveals the large variability of protocols, participants with different characteristics, and the methodological limitations evident in most studies. Some of them were conducted with less than 10 participants or with a lack of blinding procedures, while significant drop-out rates during postintervention or follow-up may have been an additional reason for the observed high risk of bias.

The results after sensitivity analysis concluded on four high-quality studies. Two of these studies used a high-intensity treatment protocol, and the other two used a low and moderate one. The results showed statistical significance (that, however, was not always coinciding with clinical importance) postintervention and at follow-up when follow-up was compared with preintervention values. On the contrary, when follow-up measurements were compared with postintervention, the results did not show clinical significance in almost all cases, indicating that further improvements were not achieved after the end of intervention.

Studies have used a variability of outcome measures, but almost all of them have used the MAL, as this measure forms part of the behavioral transfer package of CIMT. This means that MAL improvements are expected within any CIMT program, as it is the main goal of this intervention to encourage patients to try and use their upper limb as much as possible in order to reverse learned nonuse. Therefore, an increase in MAL is expected to occur during intervention and does not necessarily indicate long-lasting improvements. However, what may be an indicator of real-world improvements is when MAL values remain increased (or increase even more) at follow-up, and this is one reason why follow-up should always be included in CIMT studies. Among the 4 high-quality studies included in this review, none shows clinically significant retention of improvements in either MAL-AOU or MAL-QOM.

The heterogeneity of protocols, outcome measures, and population characteristics, which has been noted by other researchers [8, 17], makes it very difficult to reach more definite conclusions. Future studies should focus on comparing different intensities of mCIMT while also considering the severity of impairment and time after stroke and how these may relate to the overall effectiveness. It may also be worth considering for future reviews to include only specific categories of studies in terms of methodology, i.e., high-quality studies only, and/or in terms of patient characteristics, i.e., acute/chronic and functional level.

Most of the studies included in this review did not provide any information on the training background and experience of therapists providing CIT, which might be a factor influencing the outcomes. Moreover, future studies should provide clear reports on the home-based part of the intervention, which has been hardly described by most researchers in addition to information related to the third element of CIMT, i.e., the transfer package and how this has been incorporated within the protocol.

### 4.1. Study Limitations

A meta-analysis may have provided clearer results, but this was not possible with the methodology employed by the present study. To allow comparisons, this study analyzed only the first follow-up measurement, even though some studies included more than one; thus, some data on long-term effectiveness have been lost in this review. Furthermore, follow-up measurements took place at different times in each study. Reviewing all studies of mCIMT has provided a significant amount of information but also confusion due to the variability of methodologies compared.

### 4.2. Conclusions

Modified CIMT may be an effective intervention for persons with stroke but long-lasting results need to be further researched. This review does not provide support to the theory that the higher the intensity of treatment, the better the outcome, pointing to the fact that apart from the intensity, the actual content and structure of therapy matter. The ideal protocol intensity is yet to be confirmed, and it is very likely that this shall be different according to patient's characteristics, i.e., time since stroke and functional level. More sound methodologies in future studies may provide more reliable results.

## Figures and Tables

**Table 1:** Search keywords.

Keywords	PubMed	EBSCO	Scopus	Cochrane
(A) Stroke^∗^ OR Cerebral stroke OR Cerebrovascular disorder OR Cerebrovascular disease OR Cerebrovascular accident OR CVA OR Brain vascular accident OR Cerebral infarction OR Brain injury OR Brain hemorrhage OR Brain ischemia OR Brain infarction	482,527	4,805,959	859,032	77,088

(B) Constraint induced OR Constraint induced therapy OR Forced use therapy OR Constraint therapy OR CIT OR CI therapy OR Constraint-induced movement therapy OR CIMT OR Modified constraint induced movement therapy OR Modified constraint induced therapy OR mCIMT OR mCIT OR forced used	9,751	277,713	15,221	2,237

(C) Traditional rehabilitation OR Rehabilitation OR Conventory therapy OR Conventional therapy OR Traditional therapy OR Occupational therapy OR OT OR Physiotherapy OR Usual care OR Standard care OR Recovery OR Motion recovery OR Muscle strengthening OR intervention OR Isokinetic muscle strengthening OR Bilateral arm training OR Complementary interventions OR Post stroke care OR Stroke Treatment OR Stroke Rehabilitation OR Stroke Therapy OR Hands on therapy OR Repetitive task practice OR Sensory intervention OR Strength training OR Intensive care	1,556,083	25,220,900	3,534,599	563,479

A AND B AND C	596	2,909	1,032	523

Articles published between April 10^th^, 2010, and December 31^st^, 2021, in English	365	1,349	620	435

**Table 2:** Characteristics of included studies.

Study (reference)	Age (years)	Sample size (*N*)	Time since stroke (months)	Intervention period and treatment frequency	Outcome measures
	Mean (SD)		Mean		
Lin et al. [54]	46.40 (26.00)	5	21.5	3 weeks 5 days/week 120 mins/day	(i) Fugl-Meyer assessment upper extremity (FMA-UE) (ii) Motor activity log (MAL) (iii) Functional magnetic resonance imaging examination (FMRI)

Tariah et al. [43]	54.80 (10.90)	10	9.2	2 months 7 days/week 120 mins/day	(i) Wolf motor function test (WMFT) (ii) Motor activity log (MAL) (iii) Fugl-Meyer assessment upper extremity (FMA-UE)

Khan et al. [53]	60.40 (16.10)	13	5.2	6 weeks 3 days/week 90 mins/day	(i) Wolf motor function test (WMFT) (ii) Motor activity log (MAL) (iii) Chedoke-McMaster impairment inventory (CMII) (iv) AROM in shoulder flexion (v) Isometric strength shoulder flexion (vi) Isometric strength shoulder extension (vii) Isometric strength elbow flexion (viii) Isometric strength elbow extension

Wu et al. [56]	51.91 (11.93)	22	14.9	3 weeks 5 days/week 2 hours/day	(i) Action research arm test (ARAT) (ii) Motor activity log (MAL) (iii) Frenchay activities index (FAI) (iv) Stroke impact scale (SIS)

Brunner et al. [50]	61.00 (10.00)	14	1.6	4 weeks 4 days/week 60 mins/day	(i) Action research arm test (ARAT) (ii) Nine-hole peg test (9HPT) (iii) Motor activity log (MAL)

Huseyinsinoglu et al. [58]	49.10 (13.70)	11	10.6	2 weeks 5 days/week 1 hour/day	(i) Wolf motor function test (WMFT) (ii) Motor activity log-28 (MAL) (iii) Motor evaluation scale for arm in stroke patients (MESUPES) (iv) Functional independence measure (FIM)

Smania et al. [41]	63.93 (9.56)	30	11.1	2 weeks 5 days/week 120 mins/day	(i) Wolf motor function test (WMFT) (ii) Motor activity log (MAL) (iii) Ashworth scale (AS)

Treger et al. [40]	62.00 (10.40)	9	1.3	2 weeks 5 days/week 1 hour/day	(i) Number of repetitions in peg transfer, ball grasping, and “eating” with a spoon (ii) Functional independence measure (FIM) (iii) National Institute of Health Stroke Scale (NIHSS) (iv) Manual function test (MFT)

Wu et al. [55]	56.30 (12.20)	19	13.7	3 weeks 5 days/week 2 hours/day	(i) Wolf motor function test (WMFT) (ii) Motor activity log (MAL)

Wu et al. [57]	54.87 (10.24)	15	15.00	3 weeks 5 days/week 2 hours/day	(i) Kinematic analysis (ii) Fugl-Meyer assessment upper extremity (FMA-UE) (iii) Motor Activity Log (MAL)

De Diego et al. [52]	61.90 (9.70)	12	> 6 months	8 weeks 2 days/week 60 mins/day	(i) Fugl-Meyer assessment upper extremity (FMA-UE) (ii) Motor activity log (MAL) (iii) Stroke impact scale version 16 (SIS-16)

Singh and Pradhan [37]	55.20 (9.27)	20	< 1 month	2 weeks 5 days/week 120 days/week	(i) Wolf motor function test (WMFT) (ii) Fugl-Meyer assessment upper extremity (FMA-UE)

Takebayashi et al. [42]	Group 1: 53.6 (12.7)	11	15.2	2 weeks 5 days/week 5 hours/days	(i) Fugl-Meyer assessment upper extremity (FMA-UE) (ii) Motor activity log (MAL)
	Group 2: 52.0 (14.4)	10	8.9		

Van Delden et al. [47]	59.80 (13.80)	22	2.3	6 weeks 3 days/week 60 mins/day	(i) Action research arm test (ARAT)

Bang et al. [45]	56.11 (5.26)	9	16.8	4 weeks 5 days/week 60 mins/day	(i) Action research arm test (ARAT (ii) Fugl-Meyer assessment upper extremity (FMA-UE) (iii) Modified Barthel index (mBI) (iv) Motor activity log (MAL)

Barzel et al. [32]	62.55 (13.73)	85	56.6	4 weeks 10 hours/week	(i) Motor activity log (MAL) (ii) Wolf motor function test (WMFT) (iii) Stroke impact scale hand function (SIS) (iv) Nine-hole peg test (NHPT) (v) Barthel index (BI) (vi) Instrumental activities of daily living (IADL)

El-Helow et al. [31]	53.90 (7.26)	30	< 1 month	2 weeks 5 days/week 120 mins/day	(i) Fugl-Meyer assessment upper extremity (FMA-UE) (ii) Action research arm test (ARAT) (iii) Resting motor threshold (RMT) (iv) Motor evoked potential (v) Central motor conduction time

McNulty et al. [26]	56.10 (17.00)	20	6.5	2 weeks 5 days/week 60 mins/day	(i) Wolf motor function (WMFT) (ii) Motor activity log (MAL) (iii) Fugl-Meyer assessment upper extremity (FMA-UE) (iv) Box and block test (BBT) (v) Grooved pegboard

Thrane et al. [51]	65.30 (8.00)	24	< 1 month	2 weeks 5 days/week 3 hours/days	(i) Wolf motor function test (WFMT) (ii) Fugl-Meyer assessment upper extremity (FMA-UE) (iii) Nine-hole peg test (NHPT) (iv) Arm use ratio (v) Stroke impact scale (SIS)

Bang [44]	58.22 (5.17)	10	2.6	4 weeks 5 days/week 60 mins/day	(i) Action research arm test (ARAT (ii) Fugl-Meyer assessment upper extremity (FMA-UE) (iii) Modified Barthel index (mBI) (iv) Motor activity log (MAL) (v) Modified Ashworth scale (mAS)

Kwakkel et al. [46]	58.97 (14.05)	29	< 1 month	3 weeks 5 days/week 60 mins/day	(i) Action research arm test (ARAT) (ii) Fugl-Meyer assessment upper extremity (FMA-UE) (iii) Wolf motor function test (WMFT) (iv) Motricity index of the upper extremity (MI-UE) (v) Erasmus modification of the Nottingham sensory assessment of the upper extremity (EmNSA-UE) (vi) Nine-hole peg test (NHPT) (vii) Frenchay arm test (FAT) (viii) Motor activity log (MAL) (ix) Hand domain of the stroke impact scale (SIS-hand, version 3.0)

Otadi et al. [39]	50.80 (9.07)	6	3.6	3 weeks 5 days/week 120 mins/day	(i) Motor activity log (MAL) (ii) Wolf motor function test (WMFT) (iii) Modified Ashworth scale (MAS)

Shah et al. [36]	64.60 (11.70)	20	< 6 months	2 weeks 7 days/week 3 hours/day	(i) Nine-hole peg test (NHPT) (ii) Motor activity log (MAL) (iii) Fugl-Meyer assessment upper extremity (FMA-UE)

Trinh et al. [27]	55.50 (17.40)	17	10.94	2 weeks 5 days/week 60 mins/day	(i) Wolf motor function (WMFT) (ii) Motor activity log (MAL)

Yadav et al. [38]	47.03 (13.76)	30	10	4 weeks 3 days/week 3 hours/day	(i) Fugl-Meyer assessment (FMA) (ii) Motor activity log (MAL)

Bhattacharjee et al. [34]	N/I	15	N/I	2 weeks 5 days/week 30 mins/day	(i) Sollerman hand function test (ii) Wrist flexion ROM (iii) Wrist extension ROM

Yu et al. [30]	58.54 (9.61)	13	< 1 month	2 weeks 5 days/week 3 hours/day	(i) Wolf motor function test (WMFT) (ii) Motor activity log (MAL) (iii) Transcranial magnetic stimulation (TMS)

Abdullahi [49]	54.62 (6.00)	13	< 1 month	4 weeks 5 days/week 3 hours/day	(i) Fugl-Meyer assessment upper extremity (FMA-UE) (ii) Wolf motor function test (WMFT) (iii) Motor activity log (MAL) (iv) Upper limb self-efficacy test (UPSET)

Baldwin et al. [25]	59.20 (13.10)	10	8.3	2 weeks 6 days/week 60 mins/day	(i) Wolf motor function test (WMFT) (ii) Motor activity log (MAL) (iii) Adherence logbook

Doussoulin et al. [29]	Group 1: 58.33 (10.38)	24	> 6 months	2 weeks 5 days/week 3 hours/day	(i) Motor activity log (MAL) (ii) Action research arm test (ARAT)
	Group 2: 48.75 (18.60)	12			

Abba et al. [48]	59.53 (9.92)	15	7.9	6 weeks 3 days/week 45 mins/day	(i) Fugl-Meyer assessment upper extremity (FMA-UE) (ii) Modified Ashworth scale

Bhardwaj et al. [33]	Group 1: 50.0 (9.7)	16	N/I	2 weeks 6 days/week 2 hours/day	(i) Box and block test (ii) Patient-rated wrist hand evaluation score
	Group 2: 46.22 (12.48)	18			

Gauthier et al. [23]	62 (13)	40	58.8	3 weeks 10 sessions 3.5 hours/session	(i) Motor activity log (MAL) (ii) Wolf motor function test (WMFT)

Kaviraja et al. [35]	N/I	15	N/I	4 weeks 5 days/week 30 mins/day	(i) Fugl-Meyer assessment upper extremity (FMA-UE) (ii) Upper extremity functional index scale

Rocha et al. [28]	59.66 (10.04)	15	45.7	8 weeks 3 days/week 60 mins/day	(i) Fugl-Meyer motor assessment physical performance scale (FMA) (ii) Functional reach test (FRT) (iii) Stroke specific quality of life scale (SS-QOL)

Uswatte et al. [24]	55.3 (48.1–62.5)	12	28.8	2 weeks 5 days/week 3.5 hours/day	(i) Motor activity log (MAL) (ii) Wolf motor function test (WMFT) (iii) Participant opinion survey (POS)

N/I: information was not given by the authors.

**Table 3:** Treatment intensity and outcome measurements.

Study (reference)	Assessment tool	Pretreatment (mean ± SD)	Posttreatment (mean ± SD)	Follow-up (mean ± SD)	*P* value (pre-post)	*P* value (pre-follow up)	*P* value (post-follow-up)	MCID (yes/no)	Treatment time/splint wearing time
Abba et al. [48]	FMA-UE	36.8 ± 7.63	47.3 ± 7.46		Sign.			Yes	Low/low

De Diego et al. [52]	FMA-UE	24.3 ± 4.6	29.4 ± 4.9		Sign.			No	Low/low
	MAL-QOM	0.88 ± 0.34	1.39 ± 0.44		N/S			No	
	MAL-AOU	1.09 ± 0.35	1.36 ± 0.42		Sign.			No	

Kwakkel et al. [46]	ARAT	23.93 ± 13.90	45.21 ± 9.94	49.46 ± 7.46	Sign.	Sign.	N/R	Yes^a^, yes^b^, and no^c^	Low/low
	FMA-UE	42.93 ± 14.60	58.79 ± 7.17	60 ± 7.21	N/S	N/S	N/R	Yes^a^, yes^b^, and no^c^	
	MI-UE	66.21 ± 17.55	86.30 ± 12.09	86.70 ± 12.45	N/S	N/S	N/R	—	
	SIS-HF	8.83 ± 5.17	18.44 ± 5.92	21.11 ± 3.41	N/S	N/S	N/R	—	
	WMFT-PT	54.57 ± 58.69	51.94 ± 57.23	49.79 ± 56.29	N/S	N/S	N/R	No^a^, no^b^, and no^c^	
	WMFT-FA	2.38 ± 1.39	4.25 ± 0.75	4.39 ± 0.78	Sign.	N/S	N/R	Yes^a^, yes^b^, and no^c^	
	MAL-QOM	0.75 ± 0.71	2.19 ± 1.05	2.54 ± 1.03	N/S	N/S	N/R	Yes^a^, yes^b^, and no^c^	
	MAL-AOU	0.96 ± 0.76	2.52 ± 1.15	2.86 ± 1.17	N/S	N/S	N/R	Yes^a^, yes^b^, and no^c^	
	NHPT	0.10 ± 0.16	0.36 ± 0.24	0.42 ± 0.20	N/S	N/S	N/R	—	

Bang [44]	ARAT	26.40 ± 2.41	36.50 ± 3.03		Sign.			No^a^	Low/moderate
	FMA-UE	35.80 ± 3.39	45.30 ± 3.71		Sign.			No^a^	
	MAL-AOU	1.92 ± 0.25	2.73 ± 0.13		Sign.			No^a^	
	MAL-QOM	2.07 ± 0.21	2.98 ± 0.15		Sign.			No^a^	

Bang, et al., 2015 [45]	ARAT	26.00 (24.00–28.50)^d^	39.00 (34.00–40.50)^d^		Sign.			No^a^	Low/moderate
	FMA-UE	39.00 (36.50–47.00)^d^	46.00 (44.00–50.00)^d^		Sign.			No^a^	
	MAL-AOU	1.68 (1.19–1.79)^d^	2.73 (2.59–2.86)^d^		Sign.			Yes^a^	
	MAL-QOM	1.72 (1.34–1.92)^d^	2.85 (2.72–3.04)^d^		Sign.			Yes^a^	

Bhattacharjee et al. [34]	SHFT	0.73 ± 0.70	2.93 ± 0.80		Sign.			—	Low/moderate

Brunner et al. [50]	ARAT	26.46 ± 13.18	39.69 ± 13.40	44.23 ± 15.08	Sign.	Sign.	N/R	No^a^, yes^b^, and no^c^	Low/Moderate
	NHPT	0.04 ± 0.06	0.15 ± 0.17	0.22 ± 0.23	Sign.	Sign.	N/R	—	
	MAL-AOU	1.64 ± 1.16	2.92 ± 1.89	3.40 ± 1.36	Sign.	Sign.	N/R	Yes^a^, yes^b^, and no^c^	
	MAL-QOM	1.58 ± 1.18	2.80 ± 1.24	3.27 ± 1.32	Sign.	Sign.	N/R	Yes^a^, yes^b^, and no^c^	

El-Helow et al. [31]	FMA-UE	32.28 ± 9.74	49.74 ± 7.59		Sign.			Yes^a^	Low/moderate
	ARAT	31.74 ± 9.49	49.85 ± 5.27		Sign.			Yes^a^	
Kaviraja et al. [35]	FMA-UE	25.73 ± 7.01	42.13 ± 5.4		Sign.			Yes^a^	Low/moderate

Treger et al. [40]	MFT	10.2 ± 21.6	N/R	15.6 ± 22	N/R	N/S	N/R	—	Low/moderate
	Peg transfer	4.1 ± 2.4	N/R	9.1 ± 1.9	N/R	Sign.	N/R	—	
	Ball grasping	7.6 ± 2.1	N/R	13.5 ± 3.6	N/R	Sign.	N/R	—	
	Eating with a spoon	1.7 ± 0.5	N/R	3.0 ± 0.6	N/R	Sign.	N/R	—	

Van Delden et al. [47]	ARAT	23.8 ± 16.8	38.4 ± 28.8	42.9 ± 35.9	Sign.	N/R	Sign.	Yes^a^, yes^b^, and no^c^	Low/moderate
	FMA-UE	43.0 ± 14.7	50.8 ± 24.1	53.2 ± 28.1	Sign.	N/R	Sign.	No^a^, yes^b^, and no^c^	
	NHPT	0.0 ± 0.0	0.2 ± 0.2	0.0 ± 0.1	Sign.	N/R	N/S	—	
	MAL-AOU	1.4 ± 0.9	2.7 ± 2.2	2.6 ± 2.7	Sign.	N/R	N/S	Yes^a^, yes^b^, and no^c^	
	MAL-QOM	1.0 ± 0.7	2.3 ± 1.7	2.4 ± 2.4	Sign.	N/R	N/S	Yes^a^, yes^b^, and no^c^	
	SIS-HF	22.7 ± 20.0	55.3 ± 32.3	0.3 ± 19.2	Sign.	N/R	N/S	—	

Baldwin et al. [25]	WMFT-PT	59.55 ± 58.69	51.94 ± 57.23	49.79 ± 37.03	N/S	N/S	N/R	No^a^, no^b^, and no^c^	Low/high
	WMFT-FA	3.5 ± 0.8	3.8 ± 0.9	3.8 ± 0.8	N/S	Sign.	N/R	No^a^, no^b^, and no^c^	
	MAL-QOM	2.0 ± 0.9	3.3 ± 0.6	3.4 ± 0.8	Sign.	Sign.	N/R	Yes^a^, yes^b^, and no^c^	
	MAL-AOU	1.8 ± 0.9	3.3 ± 0.8	3.2 ± 0.8	Sign.	Sign.	N/R	Yes^a^, yes^b^, and no^c^	

McNulty et al. [26]	WMFT-PT	13.46 (6.7–24.53)^d^	9.97 (5.47–20.08)^d^	9.97 (4.9–18.17)^d^	Sign.	N/R	N/S	Yes^a^, yes^b^, and no^c^	Low/high
	MAL-QOM	1.6 (1.6–1.92)^d^	1.96 (1.88–2.03)^d^	1.94 (1.8–2.02)^d^	Sign.	N/R	Sign.	No^a^, no^b^, and no^c^	
	FMA-UE	40.45 (36.60–54.60)^d^	44.7 (36.60–54.60)^d^	49.4 (29.96–73.70)^d^	Sign.	N/R	Sign.	No^a^, no^b^, and no^c^	
	Box and block test	2.4 (1.7–3.2)^d^	2.8 (2.2–3.4)^d^	2.8 (2.1–3.4)^d^	Sign.	N/R	N/S	—	
	Grooved pegboard	5.0 (4.7–5.3)^d^	5.0 (4.6–5.3)^d^	5.2 (4.8–5.5)^d^	N/S	N/R	N/S	—	

Singh and Pradhan [37]	WMFT	28 ± 6.58	13.6 ± 2.86		Sign.			—	Low/high
	FMA-UE	31.15 ± 6.37	55.7 ± 6.43		Sign.			Yes^a^	

Smania et al. [41]	WMFT-FA	2.81 ± 0.87	3.62 ± 0.78	4.05 ± 0.53	Sign.	Sign.	N/R	No^a^, yes^b^, and no^c^	Low/high
	WMFT-PT	11.16 ± 15.29	4.82 ± 6.13	3.14 ± 1.53	Sign.	Sign.	N/R	Yes^a^, yes^b^, and yes^c^	
	MAL-AOU	1.45 ± 0.79	2.85 ± 0.88	3.04 ± 0.85	Sign.	Sign.	N/R	Yes^a^, yes^b^, and no^c^	
	MAL-QOM	1.41 ± 0.82	2.64 ± 0.82	2.83 ± 0.85	Sign.	Sign.	N/R	Yes^a^, yes^b^, and no^c^	

Trinh et al. [27]	WMFT-PT	17.5 (5.2–74.0)^d^	11.4 (3.0–65.3)^d^		Sign.			Yes^a^	Low/high
	MAL-QOM	1.6 (1.65–1.91)^d^	1.9 (1.66–2.03)^d^		Sign.			No^a^	

Khan et al. [53]	WMFT-PT	64.5 ± 38.4	33.0 ± 34.7	27.9 ± 29.1	Sign.	Sign.	N/R	Yes^a^, yes^b^, and no^c^	Moderate/low
	WMFT-FA	2.0 ± 1.4	3.1 ± 1.5	3.5 ± 1.4	Sign.	Sign.	N/R	Yes^a^, yes^b^, and no^c^	
	MAL-AOU	1.0 ± 1.2	2.7 ± 1.6	2.5 ± 1.9	Sign.	Sign.	N/R	Yes^a^, yes^b^, and no^c^	
	MAL-QOM	0.9 ± 1.1	2.5 ± 1.6	2.4 ± 1.9	Sign.	Sign.	N/R	Yes^a^, yes^b^, and no^c^	

Rocha et al. [28]	FMA-UE	43.20 ± 11.40	58.6 ± 2.05		Sign.			Yes^a^	Moderate/low

Bhardwaj et al. [33]	Box and block test^1^	9.12 ± 6.90	15.25 ± 9.94		Sign			—	Moderate/moderate
	Box and block test^2^	11.44 ± 8.27	17.66 ± 12.03		Sign.			—	

Lin et al. [54]	FMA-UE	55.6 ± 6.5	61.2 ± 3.9		Sign.			No^a^	Moderate/moderate
	MAL-AOU	1.9 ± 2.0	3.4 ± 1.3		Sign.			Yes^a^	
	MAL-QOM	1.9 ± 1.8	3.6 ± 1.3		Sign.			Yes^a^	

Otadi et al. [39]	MAL-QOM	0.61 ± 0.43	1.85 ± 0.59	2.33 ± 0.78	Sign.	N/R	Sign.	Yes^a^, yes^b^, and no^c^	Moderate/moderate
	MAL-AOU	0.56 ± 0.42	1.71 ± 0.55	2.30 ± 0.87	Sign.	N/R	Sign.	Yes^a^, yes^b^, and yes^c^	
	WMFT-PT	12.16 ± 3.20	9.48 ± 1.58	6.83 ± 1.32	Sign.	N/R	Sign.	Yes^a^, yes^b^, and yes^c^	
	WMFT-FA	2.8 ± 1.3	4.00 ± 0.74	4.40 ± 0.97	Sign.	N/R	Sign.	Yes^a^, yes^b^, and no^c^	

Wu et al. [55]	ARAT	35.8 ± 16.5	42.8 ± 12.8		Sign.			No^a^	Moderate/moderate
	MAL-AOU	0.6 ± 0.6	1.5 ± 0.8		Sign.			No^a^	
	MAL-QOM	0.7 ± 0.7	1.7 ± 0.9		Sign.			Yes^a^	
	SIS-HF	2.0 ± 0.8	2.6 ± 0.8		Sign.			—	

Wu et al. [56]	WMFT-PT	8.77 ± 7.67	4.02 ± 2.49		Sign.			Yes^a^	Moderate/moderate
	WMFT-FA	3.26 ± 0.65	3.78 ± 0.71		Sign.			No^a^	
	MAL-AOU	1.02 ± 0.82	2.11 ± 1.05		Sign.			Yes^a^	
	MAL-QOM	1.06 ± 0.83	2.30 ± 1.01		Sign.			Yes^a^	

Wu et al. [57]	FMA-UE	46.60 ± 8.96	50.87 ± 7.78		Sign.			No^a^	Moderate/moderate
	MAL-AOU	1.11 ± 0.74	2.06 ± 0.92		Sign.			No^a^	
	MAL-QOM	1.32 ± 0.79	2.24 ± 0.88		Sign.			No^a^	

Yadav et al. [38]	FMA-UE	34.67 ± 3.55	48.1 ± 5.42	50.57 ± 4.97	Sign.	Sign.	Sign.	Yes^a^, yes^b^, and no^c^	Moderate/moderate
	MAL-AOU	1.06 ± 0.33	1.25 ± 0.39	1.29 ± 0.42	Sign.	Sign.	Sign.	No^a^, no^b^, and no^c^	
	MAL-QOM	1.01 ± 0.31	1.22 ± 0.43	1.26 ± 0.42	Sign.	Sign.	Sign.	No^a^, no^b^, and no^c^	

Yu et al. [30]	WMFT-FA	2.53 ± 1.08	4.47 ± 0.24	4.71 ± 0.12	Sign.	N/R	N/R	Yes^a^, yes^b^, and no^c^	Moderate/moderate
	WMFT-PT	37.23 ± 34.82	12.87 ± 14.12	3.67 ± 1.44	Sign.	N/R	N/R	Yes^a^, yes^b^, and yes^c^	
	MAL-AOU	0.27 ± 0.30	2.87 ± 1.72	3.23 ± 0.67	Sign.	N/R	N/R	Yes^a^, yes^b^, and no^c^	
	MAL-QOM	0.28 ± 0.30	1.76 ± 0.61	3.44 ± 1.20	Sign.	N/R	N/R	Yes^a^, yes^b^, and yes^c^	

Doussoulin et al. [29]	MAL-AOU^3^	1.79 ± 0.95	3.09 ± 0.89	2.93 ± 0.86	Sign.	N/R	Sign.	Yes^a^, yes^b^, and no^c^	Moderate/high
	MAL-AOU^4^	1.51 ± 0.99	2.69 ± 0.88	2.53 ± 0.87	Sign.	N/R	Sign.	Yes^a^, yes^b^, and no^c^	
	ARAT^3^	37.96 ± 13.07	42.91 ± 7.75	42.37 ± 8.38	Sign.	N/R	Sign.	No^a^, no^b^, and no^c^	
	ARAT^4^	25.25 ± 17	40.19 ± 7.76	40.60 ± 8.39	Sign.	N/R	Sign.	Yes^a^, yes^b^, and no^c^	

Gauthier et al. [23]	MAL-QOM	1.50 ± 1.00	3.2 ± 1	2.6 ± 1.2	Sign.	Sign.	N/R	Yes^a^, yes^b^, and yes^c^	Moderate/high
	WMFT-PT	66.06 ± 11.74	27.54 ± 9.33	31.62 ± 9.54	Sign.	Sign.	N/R	Yes^a^, yes^b^, and no^c^	

Huseyinsinoglu et al. [58]	MAL-QOM	0.79 ± 0.58	3 ± 0.56		Sign.			Yes^a^	Moderate/high
	MAL-AOU	0.9 ± 0.6	3.2 ± 0.57		Sign.			Yes^a^	
	WMFT-FA	3.27 ± 0.96	4.03 ± 0.78		Sign.			Yes^a^	
	WMFT-PT	25.6 ± 19	15.2 ± 13.7		Sign.			Yes^a^	
	MESUPES	43 ± 7.4	48.7 ± 7.1		Sign.			—	

Uswatte et al. [24]	MAL-QOM	0.90 (0.5–1.4)^d^	3.6 (2.7–4.5)^d^	2.9 (1.9–4)^d^	Sign.	Sign.	N/R	Yes^a^, yes^b^, and no^c^	Moderate/high
	WMFT-PT	2.22 (1.71–3)^d^	1.96 (1.44–2.91)^d^		Sign.			—	

Thrane et al. [51]	WMFT-PT	13.80 ± 30	11.5 ± 28.9	12 ± 29.2	Sign.	N/R	Sign.	Yes^a^, no^b^, and no^c^	Moderate/high
	WMFT-FA	3.3 ± 0.8	4.1 ± 0.7	4.4 ± 0.8	Sign.	N/R	Sign.	No^a^, yes^b^, and no^c^	
	FMA – UE	49.4 ± 11.8	56.9 ± 10	61.6 ± 5	Sign.	N/R	Sign.	No^a^, yes^b^, and no^c^	
	NHPT	0.16 ± 0.16	0.29 ± 0.18	0.4 ± 0.19	Sign.	N/R	Sign.	—	
	SIS-HF	N/R	N/R	85 ± 15.7	Sign.	N/R	Sign.	—	

Tariah et al. [43]	WMFT-PT	16.7 ± 14.5	10.25	7.86	Sign.	Sign.	N/R	Yes^a^, yes^b^, and yes^c^	High/low
	WMFT-FA	2.96 ± 0.43	3.62 ± 0.49	3.82 ± 0.44	Sign.	Sign.	N/R	No^a^, no^b^, and no^c^	
	MAL-AOU	1.61 ± 0.88	2.97 ± 1.2	3.44 ± 1.15	Sign.	Sign.	N/R	Yes^a^, yes^b^, and no^c^	
	MAL-QOM	1.54 ± 0.78	2.78 ± 1.07	3.14 ± 1.13	Sign.	Sign.	N/R	Yes^a^, yes^b^, and no^c^	
	FMA-UE	46.7 ± 12.64	55.8 ± 6.46	54.5 ± 9.53	Sign.	Sign.	N/R	No^a^, no^b^, and no^c^	

Barzel et al. [32]	MAL-QOM	1.19 ± 1.02	1.78 ± 1.13	1.73 ± 1.28	Sign.	Sign.	N/R	No^a^, no^b^, and no^c^	High/moderate
	MAL-AOU	1.02 ± 0.97	1.58 ± 1.18	1.57 ± 1.31	Sign.	Sign.	N/R	No^a^, no^b^, and no^c^	
	WMFT-PT	17.03 ± 2.20	12.71 ± 1.62	13.37 ± 1.95	Sign.	Sign.	N/R	Yes^a^, yes^b^, and no^c^	
	WMFT-FA	2.71 ± 0.98	2.88 ± 0.96	2.88 ± 1.11	Sign.	N/S	N/R	No^a^, no^b^, and no^c^	
	NHPT	0.15 ± 0.20	0.17 ± 0.21	0.16 ± 0.21	N/S	N/S	N/R	—	
	SIS-HF	28.40 ± 28.62	39.09 ± 31.03	38.35 ± 30.93	Sign.	Sign.	N/R	—	

Takebayashi et al. [42]	MAL-AOU^5^	1.33 ± 0.55	2.12 ± 0.55	2.79 ± 0.98	Sign.	Sign.	Sign.	No^a^, yes^b^, and no^c^	High/moderate
	MAL-AOU^6^	1.18 ± 0.70	1.61 ± 0.54	1.65 ± 0.68	Sign.	Sign.	N/S	No^a^, no^b^, and no^c^	
	FMA-UE^5^	48.6 ± 7.8	55.7 ± 4.5	59 ± 3.6	Sign.	Sign.	N/S	No^a^, yes^b^, and no^c^	
	FMA-UE^6^	49.1 ± 5.5	53.3 ± 4.9	52.8 ± 6	Sign.	Sign.	N/S	No^a^, no^b^, and no^c^	

Abdullahi [49]	FMA-UE	34.00 ± 14.15	44.69 ± 9.98	50.69 ± 8.60	Sign.	Sign.	Sign.	Yes^a^, yes^b^, and no^c^	High/high
	MAL-QOM	1.62 ± 0.73	2.48 ± 0.77	3.25 ± 0.78	Sign.	Sign.	Sign	No^a^, yes^b^, and no^c^	
	MAL-AOU	1.75 ± 0.54	2.60 ± 0.76	3.40 ± 0.84	Sign.	Sign.	Sign.	No^a^, yes^b^, and no^c^	
	WMFT-FA	2.17 ± 0.82	2.68 ± 0.81	3.40 ± 0.82	N/S	N/S	Sign.	No^a^, yes^b^, and no^c^	
	UPSET	4.58 ± 2.21	6.02 ± 1.57	7.10 ± 1.42	Sign.	Sign.	N/S	—	

Shah et al. [36]	NHPT	488.5 ± 106.5	450.5 ± 105.9		N/S			—	High/high
	MAL	1.3 ± 6.5	1.93 ± 0.6		Sign.			No^a^	
	FMA	82.7 ± 10.6	90.7 ± 11.8		Sign.			—	

^1^mCIMT with interval; ^2^mCIMT without interval; ^3^group modality; ^4^individual modality; ^5^transfer package group; ^6^without transfer package group; ^a^pre to posttreatment difference; ^b^pretreatment to follow-up difference; ^c^posttreatment to follow-up difference; ^d^median (IQR); MCID: minimally clinically important difference; N/R: not reported; N/S: not significant; MI-UE: motricity index of the upper extremity; SIS-HF: hand domain of the stroke impact scale; NHPT: nine-hole peg test; SHFT: Sollerman hand function test; MFT: manual function test; MESUPES: motor evaluation scale for arm in stroke patients; UPSET: upper limb self-efficacy test.

**Table 4:** Methodologic quality of included studies.

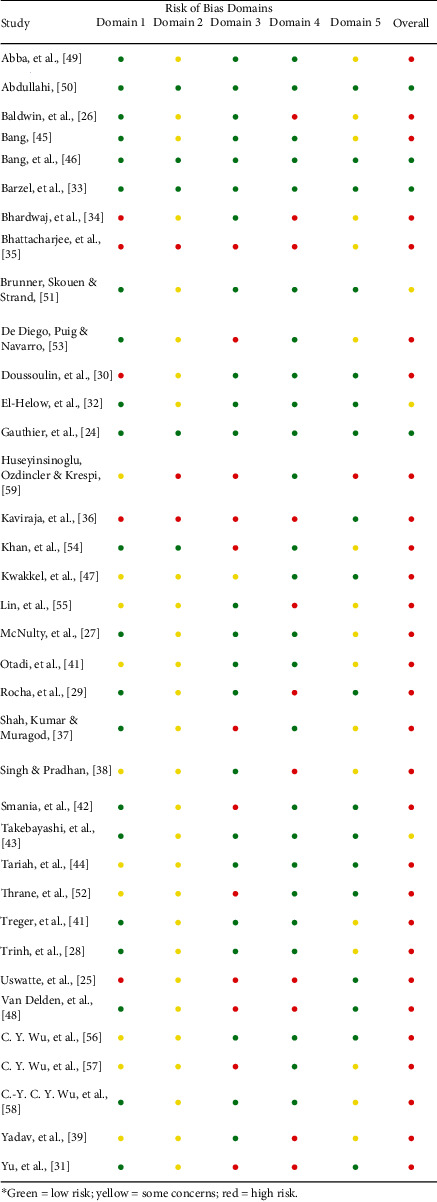	

## Data Availability

Data will be available directly by the authors upon request.

## Abbreviations

ARAT: Action research arm test
CIMT: Constraint-induced movement therapy
FMA-UE: Fugl-Meyer assessment for upper extremity
ICF: International classification of functioning, disability, and health
MAL-AOU: Motor activity log amount of use
MAL-QOM: Motor activity log quality of movement
mCIMT: Modified constraint-induced movement therapy
PRISMA: Preferred reporting items for systematic reviews and meta-analysis
RCTs: Randomized controlled trials
ROB 2: Risk-of-bias tool for randomized trials 2
SMDs: Standardised mean differences
WMFT- PT: Wolf motor function test performance time
WMFT-FA: Wolf motor function test functional ability.
